# Magnetoacoustic Imaging of Electrical Conductivity of Biological Tissues at a Spatial Resolution Better than 2 mm

**DOI:** 10.1371/journal.pone.0023421

**Published:** 2011-08-12

**Authors:** Gang Hu, Bin He

**Affiliations:** Department of Biomedical Engineering, University of Minnesota, Minneapolis, Minnesota, United States of America; Cornell University, United States of America

## Abstract

Magnetoacoustic tomography with magnetic induction (MAT-MI) is an emerging approach for noninvasively imaging electrical impedance properties of biological tissues. The MAT-MI imaging system measures ultrasound waves generated by the Lorentz force, having been induced by magnetic stimulation, which is related to the electrical conductivity distribution in tissue samples. MAT-MI promises to provide fine spatial resolution for biological tissue imaging as compared to ultrasound resolution. In the present study, we first estimated the imaging spatial resolution by calculating the full width at half maximum (FWHM) of the system point spread function (PSF). The actual spatial resolution of our MAT-MI system was experimentally determined to be 1.51 mm by a parallel-line-source phantom with Rayleigh criterion. Reconstructed images made from tissue-mimicking gel phantoms, as well as animal tissue samples, were consistent with the morphological structures of the samples. The electrical conductivity value of the samples was determined directly by a calibrated four-electrode system. It has been demonstrated that MAT-MI is able to image the electrical impedance properties of biological tissues with better than 2 mm spatial resolution. These results suggest the potential of MAT-MI for application to early detection of small-size diseased tissues (e.g. small breast cancer).

## Introduction

Electrical impedance imaging approaches have been widely explored for many years stemming from the fact that changes in electrical impedance values of biological tissues are related to their physiological and pathological status, and that this relationship may provide useful information for clinical applications. For example, experimental results have indicated that malignant breast tumors exhibit lower electrical resistivity (higher electrical conductivity) than the surrounding normal tissues [1]–[3]. Clinical data from 24,740 cases suggest that five-year breast cancer survival rate can reach 96.2% if the tumor diameter is diagnosed when less than 5 mm [4]. For these reasons, increased attention has been paid to the development of high spatial resolution imaging techniques for the early detection of small-size tumor. One of the existing methods is electrical impedance tomography (EIT) [5]–[7] – an imaging method that produces impedance images with good contrast and good temporal resolution for dynamic bioimpedance imaging. However, the spatial resolution of EIT is low, due to the nonlinear nature of the ill-posed inverse problem, limiting its applications to imaging small tumor. Magnetic resonance electrical impedance tomography (MREIT) is a recently introduced technique that combines MRI and EIT techniques [8]–[10]. MREIT uses the MR scanner to measure the magnetic field disturbance caused by the injected current flowing in conductive tissues. MREIT is capable of providing good spatial resolution. However, in order to obtain an adequate signal-to-noise ratio (SNR), a high level of stimulating current is currently still required, which limits its clinical application. In contrast to EIT and MREIT, whereby electrical excitation is delivered through electrodes, magnetic induction tomography (MIT) [11] utilizes coils to deliver magnetic excitation and make magnetic measurements. The spatial resolution of MIT, however, is still limited as in EIT [12]. Other strategies for high resolution electrical impedance imaging, including magneto-acoustic tomography (MAT) [13], [14] and hall effect imaging (HEI) [15] utilize the coupling mechanism between the electromagnetic field and acoustic field. Compared to EIT and MREIT, MAT adopts ultrasound measurements with surface current injection, or its reverse mode as HEI, in which the vibrations created by ultrasound propagating in tissues would result in a detectable Hall voltage at the boundaries. Limitations due to current stimulation or voltage measurement still exist, for example, the shielding effects from the outer low-conductivity layers (e.g. fat) in tissues would prevent the currents from penetrating the tissues.

To overcome these limitations, a novel method termed *magnetoacoustic tomography with magnetic induction* (MAT-MI) was recently introduced by He and co-workers [16]–[20]. In MAT-MI, microsecond width pulsed magnetic excitation is delivered to induce circulating currents in the conductive medium (e.g. soft tissue), together with a static magnetic field. A Lorentz force occurs and produces the mechanical vibrations in the tissues that will emit acoustic waves. The wave signals transmit through the volume and are acquired by surrounding acoustic sensors. The tomographic conductivity distribution is then reconstructed by combining these collected acoustic signals. As the biological tissue is highly permeable to magnetic stimulation at low frequency (less than 2 MHz), the shielding effects no longer exist. In addition, microsecond-width (or even shorter) magnetic stimulation produces a peak frequency of the detected acoustic wave at several megahertz, which could generate images with submillimeter spatial resolution. While the spatial resolution is still not as high as that of conventional clinical imaging techniques such as X-ray computed tomography (CT) and magnetic resonance imaging (MRI), conventional CT or MRI does not provide the capability of imaging electrical properties. Due to the unique feature of imaging electrical properties of tissue and low cost of instrumentation, MAT-MI offers unique merits in imaging electrical properties of biological tissues.

MAT-MI experimental studies have been previously carried out on copper loops [16], gel phantoms [17] and salted tissue samples [18]. Recent studies have suggested that the MAT-MI is sensitive to image low-conductivity fresh animal tissues [19] and human diseased tissues [20] with naturally formed tumor-to-normal interface. Due to the use of acoustic measurements for image reconstruction, MAT-MI may have the potential for high resolution imaging. However, there has been no quantitative study on its exact spatial resolution. In the present study, we carry out MAT-MI acoustic source reconstruction to estimate the imaging resolution, defined as the minimum resolvable distance between two adjacent acoustic sources in a cross-sectional image. MAT-MI ultrasound signals were acquired by an unfocused transducer in a circular measurement configuration. The acoustic source distribution was then computed based on a back-projection algorithm to reflect the electrical conductivity contrast in samples. Computer simulations and experimental methods were used to determine the spatial resolution of the present MAT-MI system. Tissue-mimicking gel phantoms and fresh animal tissue samples were also investigated.

## Materials and Methods

### Principles of MAT-MI

In the MAT-MI system, a pulsed magnetic field stimulation **B**_1_(**r**, *t*) is delivered to the object through a nearby current-carrying magnetic coil. According to the Faraday's law, an induced electric field **E**(**r**, *t*) will be established in the space due to the presence of the time-varying magnetic field. The intensity of induced electric field is proportional to the rate of change of the magnetic stimulation. If the object is conductive (e.g. biological tissue) with conductivity distribution **σ**(**r**), eddy currents will appear in the object because of the induced electric field **E**(**r**, *t*). Here, the displacement current is negligible [21]. The current density distribution **J**(**r**, *t*) inside the object can be computed by Ohm's law **J**(**r**, *t*) = **σ**(**r**)**E**(**r**, *t*). In MAT-MI, the working frequency is at the MHz level. The wavelength of the electromagnetic field is much bigger than the object size. Thus, the quasi-static condition is satisfied. If the duration of the magnetic stimulation is short enough, the **J**(**r**, *t*) can be written as the product of a pure spatial function **J**(**r**) and a delta function δ(*t*). When a static magnetic field **B**_0_(**r**) is applied to the object, Lorentz force occurs as:
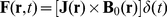
(1)The Lorentz force will cause the mechanical vibrations that can generate acoustic waves. These waves propagate through the tissues and are then collected by the surrounding acoustic detectors for image reconstruction. A wave equation in an acoustically homogenous medium can be written as [16]:
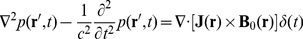
(2)where the *p*(**r′**, *t*) is the acoustic pressure at spatial point **r′**, *c* is the acoustic speed of ∼1.5 mm/µs for soft tissues. The right hand side of Eq. (2) is divergence of Lorentz force, which acts as the acoustic source. By using the Green's function, the acoustic field can be solved as [22]:
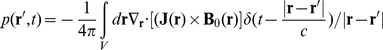
(3)Eq. (3) implies that the pressure signal at a detection point is proportional to the volumetric integration of the divergence of the Lorentz force in the object over a spherical surface. Either analytic method [23] or finite element method (FEM) [24] can be used to calculate the acoustic pressure distribution. When the measured or simulated pressure signals are known, the acoustic source distribution can be calculated using the time reversal method [16], [25].
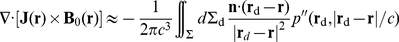
(4)where **r**_d_ is the detection point on the surface Σ, **n** denotes the normal vector at **r**_d_. *p*″ is the second derivative of the acoustic pressure over time. Eq. (4) indicates that the acoustic source distribution can be reconstructed by a back-projection algorithm based on 3D acoustic measurements on a whole detection surface surrounding the sample. When the sample has uniform structure in the direction perpendicular to the scanning plane, we can use 2D experimental data to reconstruct the cross-section image [16].
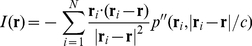
(5)Where *I*(**r**) is the acoustic source distribution, **r**_i_ is the position for the *i*th scanning point. *N* is the scanning channel number.

### MAT-MI imaging system

**Figure 1:**
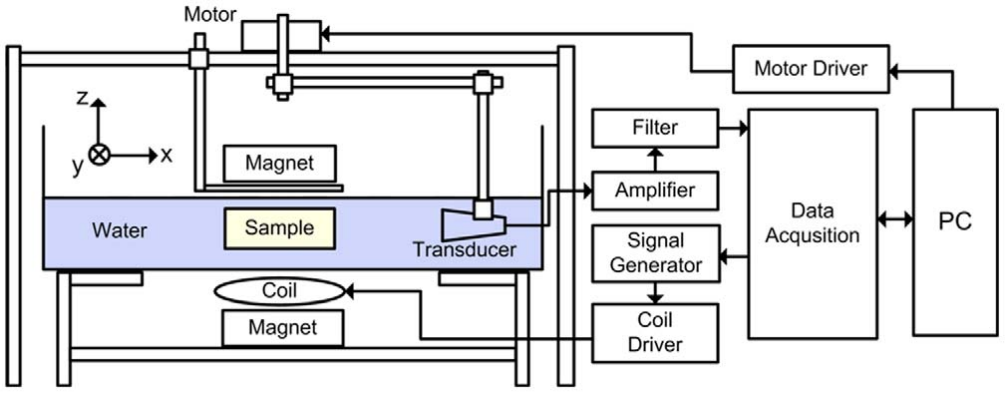
Diagram of MAT-MI hardware system setup.

Figure 1 shows the hardware setup used for MAT-MI experiments [17]–[20]. Both the sample and the transducer were immersed under distilled water for acoustic signal coupling. A 25 mm-diameter unfocused transducer (TRS Ceramics, PA, USA) was mounted on a right-angled holder driven by a step motor (B5990TS, Velmex, NY, USA) for mechanical rotation. This transducer was customized with piezocomposite material for high detection sensitivity. It had a nominal center frequency of 0.5 MHz, −6 dB fractional bandwidth of 0.71. Piezoelectric signals collected by transducer were amplified with a low-noise ultrasound amplifier (5660B, Olympus, MA, USA) and a second-stage amplifier with a built-in filter (VP2000, Reson, Denmark) before the data digitalization. A customized magnetic stimulator was employed to deliver magnetic stimulation to induce sufficient eddy currents. The trigger signal fed to the magnetic stimulator was provided by a signal generator (33220A, Agilent, CA, USA). The five-turn magnetic coil had an outer diameter of 70 mm. The coil driver was equipped with a high-voltage source, an internal capacitor and a solid state switch for handling large currents. The charging voltage was adjustable and the maximum working voltage was 24 kV. Fast discharge of the capacitor produced a pulsed current flowing through the coil.

To build a static magnetic field, two stacked NdFeB permanent magnets (DZ08-N52, K&J Magnetics, PA, USA) with diameter of 75 mm, height of 38 mm, and weight of 1.3 kg were placed on the top and bottom of the sample. The separation between the two magnets was adjustable according to the sample thickness. In this study, the measured magnetic flux density was 0.2∼0.3 Tesla. A data acquisition system (CS8324, Gage Applied, Canada) together with a personal computer was used for trigger control, signal synchronization and data transferring. A sampling rate at 5 MHz was used to record 2048 data points at each view angle. All control codes were written using Labview graphic programming language (National Instruments, TX, USA).

### Direct conductivity measurement

We made a direct conductivity measurement device to obtain the absolute electrical conductivity of biological samples. This measurement was based on the four-electrode probe technique, which has been widely used for measuring tissue impedance [26], [27]. The electrodes were made with 300 µm diameter stainless steel needles (Biopac System, CA, USA). All electrodes were coated with Teflon material for complete electrical isolation, except for the 1.5–1.8 mm conductive tip. Four electrodes were built in a line array with an inter-electrode spacing of 2 mm. The injection current was applied to the sample through a 1 k**Ω** resistor by a signal generator (33220A, Agilent, CA, USA). The signal generator was fixed with 460 kHz, 1.0 V peak-to-peak sine wave output. The electrode for sensing the sample current was connected to a current-to-voltage converter with 1 k**Ω** feedback resistor. The voltage difference signals were picked up by high input impedance (10^12^ **Ω**) differential amplifier followed by a 20 dB gain voltage amplifier. Both the differential voltage and the current passing through the sample were recorded by a digital scope (DSO7014A, Agilent, CA, USA) with 64 times data averaging. The probe constant was calibrated by measuring the standard calibration saline solution (Oakton Inc, IL, USA) at the experimental temperature. Three solutions with nominal conductivity value of 0.26 S/m, 0.83 S/m, and 1.19 S/m at 21°C were employed. The calculated probe constant was found to be stable at 0.95 cm^−1^, which indicated good linearity for soft tissue conductivity measurement.

### Spatial resolution estimation

**Figure 2:**
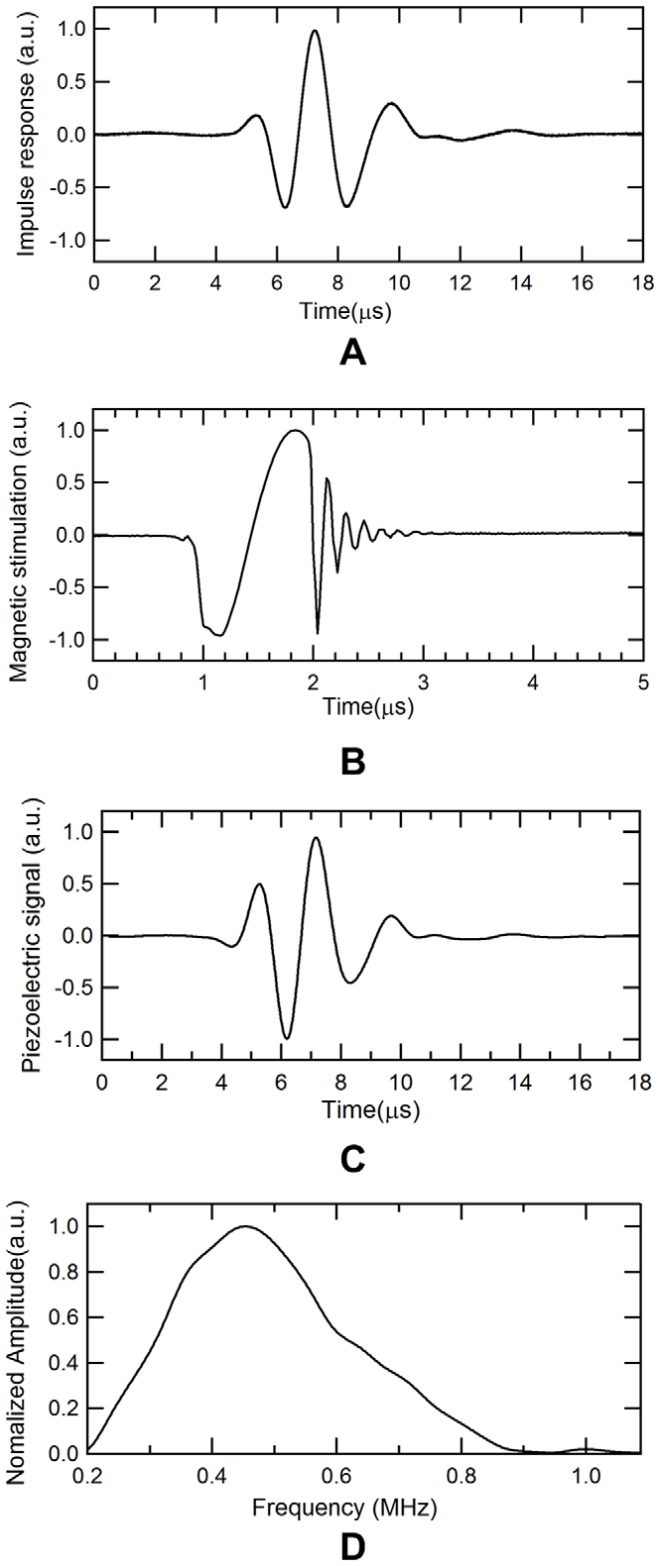
Measured system parameters for spatial resolution estimation. (A) Impulse response of the transducer. (B) Waveform of magnetic stimulation. (C) Calculated MAT-MI system response function *H*(*t*). (D) FFT spectrum of *H*(*t*).

In this study, we used a 2D circular measurement configuration to collect data from various angles (Fig. 1). The spatial resolution in the cross-sectional image is thus approximately equal to the axial resolution along the acoustic axis. When an imaging system is based on the measurements of induced ultrasound signals, the axial spatial resolution will be primarily determined by three factors [28]: the duration of excitation, the frequency response of the transducer, and the physical geometry of the transducer. When the detection radius is much bigger than the transducer diameter, we can assume the transducer as a point receiver in the space by ignoring its physical size. Therefore, the final resolution is mainly determined by the time response function of the imaging system *H*(*t*), which can be written as [29]:
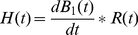
(6)where dB_1_(*t*)/d*t* is the magnetic stimulation profile and *R*(*t*) is the impulse response of transducer. * denotes the convolution. Figure 2A shows the temporal profile of the transducer impulse response used in this study. Figure 2B is the magnetic stimulation waveform obtained by recording the induced voltage signal at the twisted leads of a small sensing coil nearby the excitation coil. Figure 2C depicts the calculated MAT-MI system response function *H*(*t*) according to Eq. (6). The Fast Fourier Transform (FFT) is performed on *H*(*t*) and the spectrum data *H*(*f*) is shown in Fig. 2D. The peak frequency of *H*(*f*) is located at 460 kHz with half-amplitude bandwidth of 300 kHz. The measured MAT-MI signal *p_m_* can be written as a convolution:
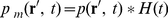
(7)where *p* (**r′**, *t*) is the full-bandwidth MAT-MI signal with a delta function input.

Here, the FWHM of the PSF can be used to estimate the spatial resolution. We firstly conduct a well-controlled numerical simulation to compute the PSF. For simplicity, the transducers in the simulation program are all placed at the plane Z = 0. The transducer is considered a point receiver due to the large detection radius compared with the small diameter of transducer. We assumed the object and its surrounding medium are acoustically homogeneous, which equates to a constant acoustic speed through the entire volume. No acoustic scattering and attenuation are considered in this simulation. In order to make a comparison to the experimental data, we set most of the parameters in the simulation program identical to those used in practical experiments. The sampling rate is 5 MHz for data acquisition and detection radius is 292 mm for circular measurement. The acoustic speed is set to be 1.5 mm/µs. We can easily generate MAT-MI ultrasound data from 360 channels with an angular step of 1° (to cover a full view angle) because no mechanical block exists in the simulation. The acoustic pressure signals are calculated by the Eq. (7) based on measured system response function. In the inverse calculation, we reconstruct the acoustic source distribution on a 30×30 mm^2^ region with grid size of 0.3×0.3 mm^2^ based on of Eq. (5).

Experiments on a gel phantom were also completed to check the actual spatial resolution. In a practical experiment, the PSF map from an ideal delta point source will not be available. However, we can use a pair of parallel line acoustic sources with variable separation to test the resolution. The phantom for this testing was made of 5% salinity gel for high image contrast. Two square gels with size of 21×18×6 mm^3^, 21×13×6 mm^3^ were surrounded by mineral oil. In order to simulate good line acoustic sources, the edges of the phantoms were made as sharp as possible by cutting them when they were still under low temperature status. When making the phantom, we incorporated more gel powder into the mixed solution to make them solid. In addition, the thickness of the phantoms was chosen to be small to minimize the deformation caused by gravity.

### Tissue-mimicking gel phantom imaging

Biological tissues usually exhibit small conductivity values, especially when the frequency of applied electrical field is low [31]. At MHz ultrasound frequency, the conductivity for most human soft tissue with high water content is 0.20∼0.70 S/m [32]. To test the applicability of MAT-MI for tissue imaging, we built a tissue-mimicking gel phantom based on the following steps. First, we added 1.5 g NaCl (Mallinckrodt Baker, NJ, USA) and 15 g gelatin (Sigma, MO, USA) to 500 g distilled water to make a mixed hot solution with salinity of 0.29%. We placed a metal cube (25×25×35 mm^3^) in a thin-wall plastic container (50 mm diameter, 30 mm height) to build a mold. Then we injected the solution into the mold carefully without introducing any air bubbles. After cooling down to the room temperature (21°C) for about 30 minutes, the solution became solid. The metal cube was removed from the container to leave a square hole inside the gel. At last, we poured the mineral oil into the hole to finalize this phantom. In the experiment, the phantom was positioned at the center of the tank. The transducer collected MAT-MI signals with a step of 2°. At each angle point, 200 times averaging was used. After the scanning, the direct conductivity measurements at ten different positions in the gel were conducted with the method mentioned above. The gel exhibited a very uniform conductivity distribution and the value was around 0.50 S/m at 460 kHz, which is comparable to human soft tissue. The conductivity of the mineral oil was close to zero.

### Biological tissue imaging

In addition to the low conductivity property, the biological tissues commonly present complex geometry and non-negligible ultrasound attenuations. Due to this fact, we also completed an experiment on a well-controlled animal tissue sample. All these tissues were purchased from a local grocery store and the sample was composed by pressing a piece of fresh goat muscle and a piece of fresh pork fat together. The muscle portion has a dimension of 18×6×10 mm^3^ and the fat portion has a dimension of 19×7×10 mm^3^. For ultrasound signal coupling, the cooled gel solution was used to fill the gap between the tissue sample and the container. Scanning with angular step of 2° was done with 200 times signal averaging at each view angle. Immediately after the scanning, the direct conductivity measurements were conducted on the two portions. Each portion included ten different measurement points. The pork fat tissue had very low conductivity around 0.01–0.03 S/m. The goat muscle showed higher conductivity value around 0.62–0.67 S/m.

## Results

**Figure 3:**
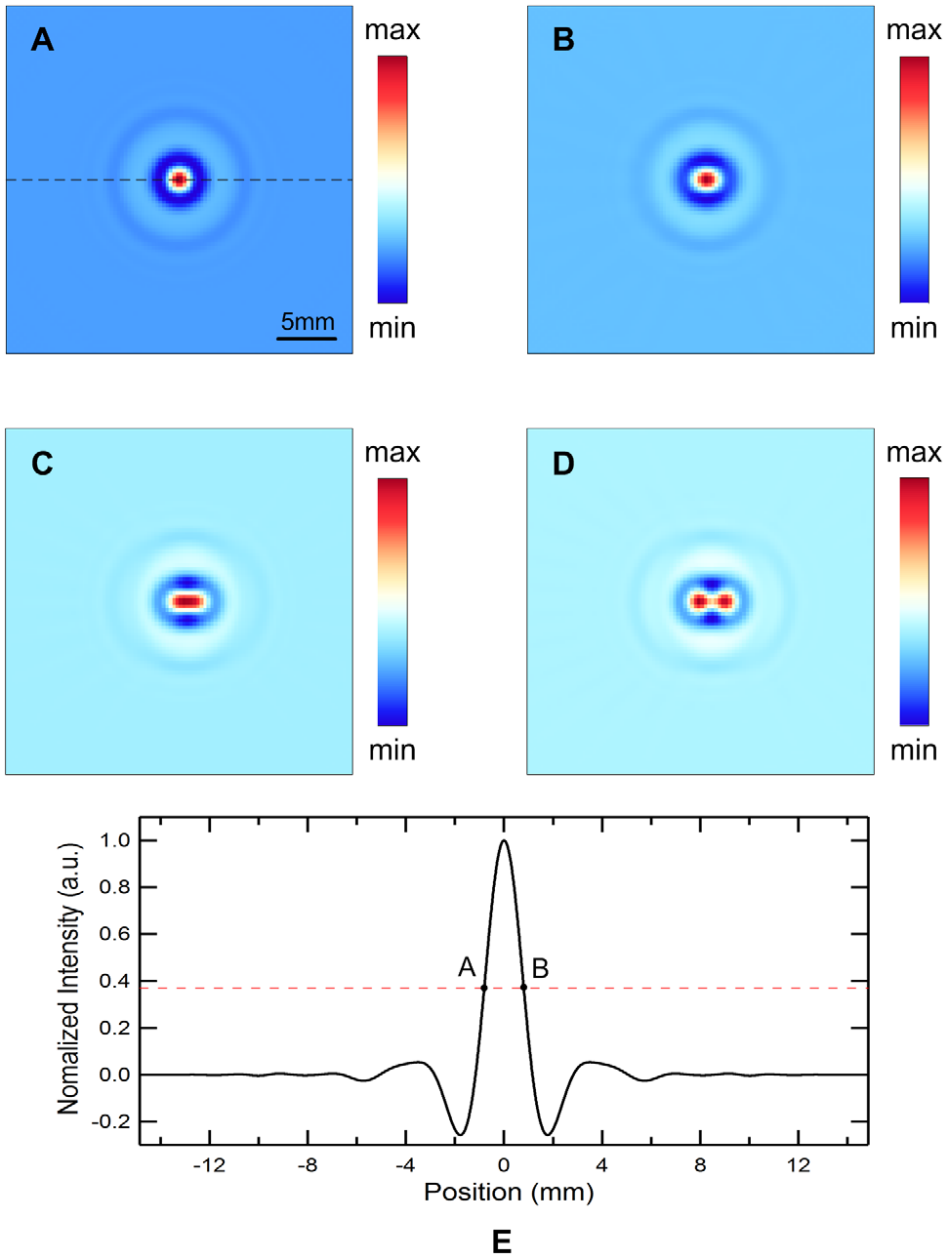
Computed MAT-MI images and intensity profile from ideal point sources. (A) Reconstructed image of a point MAT-MI acoustic source located at the origin. (B), (C) and (D) are the reconstructed MAT-MI images of two acoustic sources with a gap *d* of 1 mm, 1.5 mm, 2.0 mm, respectively. (E) The profile of the reconstructed intensities along the center dash line in (A).

Figure 3A is the reconstructed image of the single point source located at the center of the field of view, which represents the 2D PSF response of the present MAT-MI imaging system. This PSF map is radially symmetric because the point source is exactly positioned at the origin. Figure 3B–D present the reconstructed image of the two point sources with varying distances of 1 mm, 1.5 mm, and 2.0 mm, respectively. The two points still fuse together when *d* = 1.5 mm. However, they can be clearly resolved if the distance is increased to 2.0 mm. Consequently, the spatial resolution should be between 1.5 mm and 2.0 mm. For accurate estimation of the resolution, the intensity values along the center line of the PSF map in Fig. 3A are extracted and then smoothed with linear interpolation. The normalized intensity profile is shown in Fig. 3E. The half amplitude line (dashed line) corresponding to the intensity value at 0.37 intersects the profile (solid line) at point A and point B. Here, the distance |X_AB_|, which is the FWHM of PSF, is determined to be 1.62 mm for the spatial resolution of the imaging system. This value agrees well with the theoretical resolution predicted by the half wavelength of the detected ultrasound signals, which has a center frequency at 460 kHz and an acoustic speed at 1.5 mm/µs in the medium.

**Figure 4:**
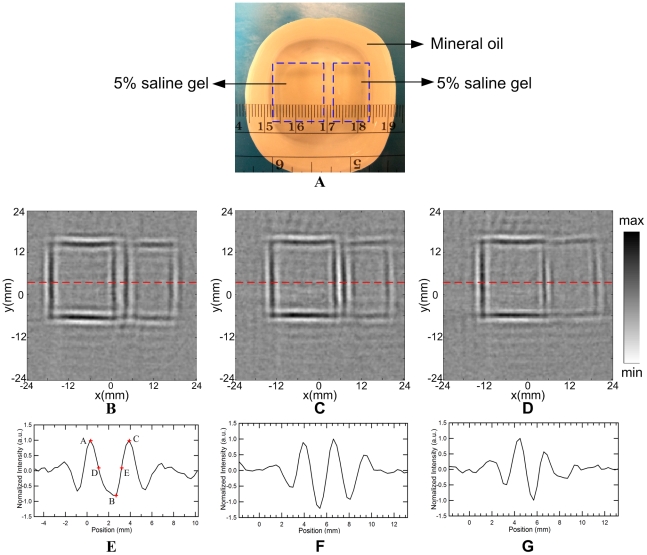
Gel phantom experiments to test actual spatial resolution. (A) Photograph of a phantom to test the spatial resolution. Two square gels with 5% salinity were immersed in mineral oil. The gap between the two gel phantoms is *d*. (B), (C) and (D) are the reconstructed MAT-MI images of phantom in (A) with a gap *d* of 3 mm, 2 mm, 1 mm, respectively. (E), (F) and (G) are the profiles of the reconstructed intensities along the dash line (Y = 3.6 mm) in (B), (C) and (D), respectively.

Figure 4B–D show the reconstructed MAT-MI images of the two square gels in Fig. 4A with variable gap *d* = 3 mm, 2 mm, 1 mm, respectively. The normalized intensity profile along the dashed lines (Y = 3.6 mm) are plotted in Fig. 4E–G. When the gap is greater than 2 mm, the two parallel lines can be distinguished. If the gap is reduced to 1 mm, the two lines can not be recognized clearly. To quantify the resolution, the two crest points at A, C and one trough point at B are firstly determined in Fig. 4E. Then, the half amplitude points D and E are found according to Y_D_ = (Y_A_+Y_B_)/2, Y_E_ = (Y_C_+Y_B_)/2. The spatial resolution of the system is estimated by Rayleigh criterion [30]. The two sources can not be resolved when point D encounters point E. Therefore, the spatial resolution *R*, the minimum discernible distance, can be approximately calculated by *R* = |X_AC_|−|X_DE_|. From Fig. 4E, the *R* is measured to be 1.51 mm. The experimental spatial resolution is slightly better than that from the computer simulation. This is mainly due to the different working mode of transducer. In the simulation, the impulse response of the transducer in Fig. 2C is obtained from the manufacturer including the transducer excitation and the eletro-mechanical impulse response during emission and reception of the pulse, which means a transmitting-receiving mode widely used in the pure ultrasound imaging. In MAT-MI imaging experiments, however, the transducer works only in a receiving only mode to collect the induced ultrasound wave propagating one way to the transducer. The wider bandwidth in transducer receiving mode would offer better resolution in reconstructed images because more high-frequency components are collected.

**Figure 5:**
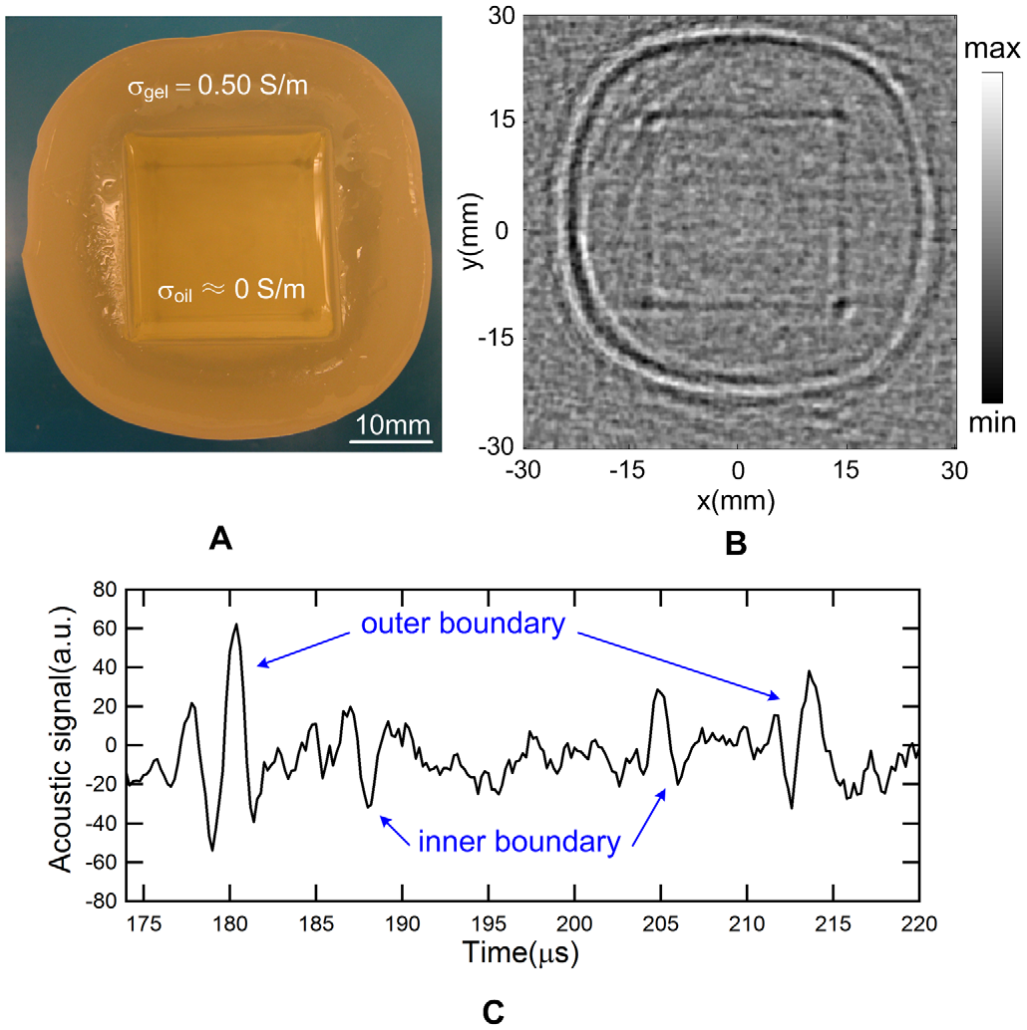
Low-conductivity tissue-equivalent gel phantom experiments. (A) Photograph of the gel phantom. The outer layer had a conductivity of 0.50 S/m. The inner square hole was filled with non-conductive mineral oil. (B) Reconstructed MAT-MI image of the phantom shown in (A). (C) Recorded temporal MAT-MI signal when the transducer was placed west to the phantom. The four bipolar-structure signals were corresponding to the four conductivity interfaces.

The reconstructed image of the phantom in Fig. 5A is shown in Fig. 5B. The boundaries between the gel and oil can be imaged, and the shape and the size as well as the location of the mineral oil have been identified with a good spatial resolution. Figure 5C shows a recorded MAT-MI signal when the transducer was exactly located at the position left to the phantom. We can see four bipolar-structure ultrasound signals appearing at different time points from the start point. Two outer structures with a delay of 33 µs indicate the edges of the plastic container with a diameter of 50 mm by considering the acoustic speed of 1.5 mm/µs in the medium. Two inner structures point out the two gel-to-oil boundaries spacing by 25 mm.

**Figure 6:**
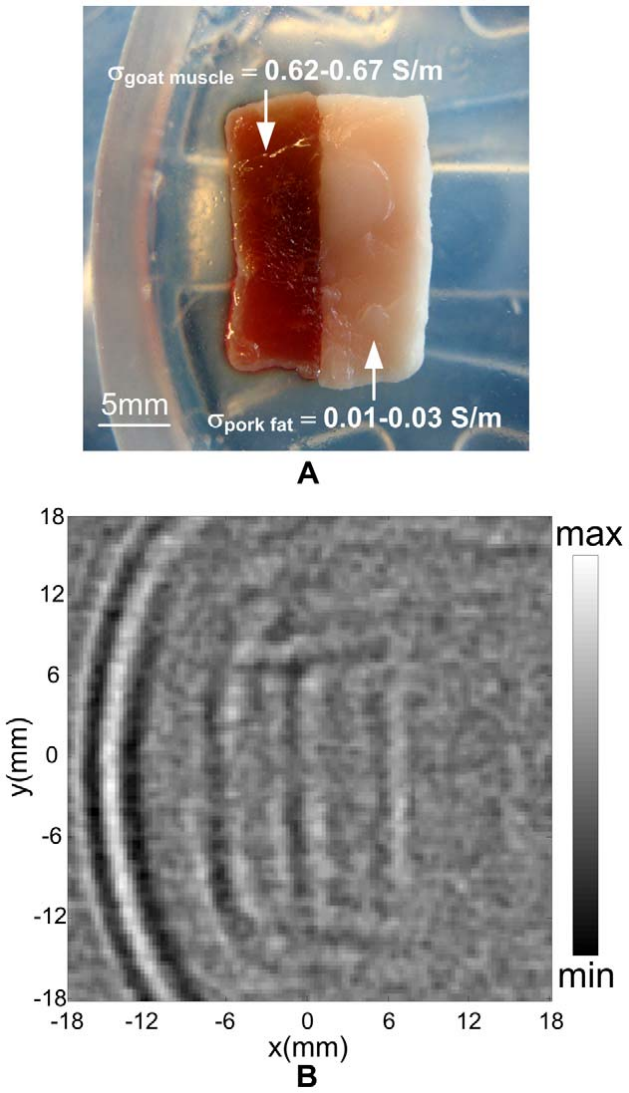
Biological tissue experiments. (A) Photograph of a fresh animal tissue sample composed of a piece of goat muscle and a piece of pork fat. (B) Reconstructed MAT-MI images of the sample shown in (A).

Figure 6B illustrates the reconstructed image agreeing roughly with the original photograph in Fig. 6A. Some deformations at the sample boundaries are observed. This is mainly due to the fact that these biological tissues are composed of a very soft structure. We only acquired MAT-MI signal in the XY plane and reconstructed the image based on the 2D form in Eq. (5). The nonuniform distribution of conductivity along the Z thickness direction would cause the image deformation according to Eq. (4). However, the sample outline including the three vertical lines can still be imaged. It has been verified by previous experiments that the tiny mechanical discontinuities at the tissue interface will not contribute much to the induced MAT-MI ultrasound signals [20]. Hence, the middle vertical line in Fig. 6B reflects a conductivity change at the muscle-to-fat tissue interface there. As our MAT-MI system resolution is tested to be better than 2 mm, the outlines of the 6 mm-width goat muscle and the 7 mm-width pork fat are both clearly imaged, thus demonstrating the ability of our MAT-MI system to image soft tissue conductivity property with millimeter resolution.

## Discussion

The MAT-MI system uses ultrasound measurements to detect acoustic sources generated by the induced Lorenz force, which is directly related to the conductivity distribution of the object. Because the ultrasound scattering in soft tissues is low, MAT-MI promises to provide bioimpedance images with spatial resolution close to pure ultrasound imaging. However, quantitative evaluation of MAT-MI imaging spatial resolution has not been reported. In this study, using both computer simulation and an experimental method, we determine the resolution of our present experimental MAT-MI imaging system to be 1.51 mm. This testing result agrees approximately with the theoretical prediction limit for an ultrasound imaging system with the working frequency centered close to 460 kHz. As discussed in this paper, the duration of the system response function will be the main factor to determine the available spatial resolution. Therefore, a better spatial resolution can be achieved by using a shorter magnetic stimulation and a higher frequency transducer. The drawback resulting from using a high frequency system, however, is the introduction of more ultrasound attenuation in tissues, which will decrease the signal-to-noise ratio (SNR) or penetration depth for the acoustic measurements. Thus, there remains a tradeoff between spatial resolution and SNR.

In this study, we use acoustic source reconstruction rather than conductivity reconstruction to electrically reveal the structure of the samples. Theoretically, for a medium with an electrically homogeneous distribution, the conductivity information can be well reconstructed when the wide-bandwidth MAT-MI signals are available [23]. However, the commercial ultrasound transducer is usually fabricated with a limited frequency bandwidth. This kind of transducer will be acting as a bandpass filter which rejects most of the out-of-band MAT-MI acoustic signals. As a consequence, the imaging system will only collect those pulse-like signals coming primarily from the conductivity boundaries because the abrupt changes at the tissue interfaces generate MAT-MI signals with relatively large bandwidth. Conversely, the low-frequency signals between the boundaries (which can reflect the conductivity itself) are hardly detectable as shown in Fig. 5C. Consequently, our present MAT-MI imaging system only gives the conductivity boundary information for biological tissues unless the lost frequency components can be well recovered. Unlike the gel phantoms or tissue samples with sharp interfaces used in this study, the conductivity distributions of the real soft tissues are much more complex. The tissue transition zone can be broad to make the conductivity changes gradual, which will result in decreased MAT-MI signal intensity due to the decreased conductivity gradient predicted by Eq. (3). Hence, the development of high-sensitivity imaging system is still needed to yield images of acceptable signal-to-noise ratio.

In conclusion, we report the quantitative analysis and examination on the spatial resolution of our present MAT-MI imaging system. Our experimental results suggest that the MAT-MI method was able to image the electrical conductivity properties of biological soft tissues with millimeter level spatial resolution. The actual resolution of our present MAT-MI system has been tested to be 1.51 mm, which shows its potentials for the detection of small-size diseased tissues (e.g. breast cancers) in their early stage of development.
